# Enrichment of statistical power for genome-wide association studies

**DOI:** 10.1186/s12915-014-0073-5

**Published:** 2014-10-17

**Authors:** Meng Li, Xiaolei Liu, Peter Bradbury, Jianming Yu, Yuan-Ming Zhang, Rory J Todhunter, Edward S Buckler, Zhiwu Zhang

**Affiliations:** College of Horticulture, Nanjing Agricultural University, Nanjing, 210095 China; Institute for Genomic Diversity, Cornell University, Ithaca, New York 14853 USA; United States Department of Agriculture (USDA) – Agricultural Research Service (ARS), Ithaca, New York 14853 USA; Department of Agronomy, Kansas State University, Manhattan, Kansas 66506 USA; State Key Laboratory of Crop Genetics and Germplasm Enhancement/National Center for Soybean Improvement, College of Agriculture, Nanjing Agricultural University, Nanjing, 210095 China; Department of Clinical Sciences, College of Veterinary Medicine, Cornell University, Ithaca, New York 14853 USA; College of Agronomy, Northeast Agricultural University, Harbin, Heilongjiang 150030 China; Department of Crop and Soil Science, Washington State University, Pullman, WA 99164 USA

**Keywords:** Genome wide association study, population structure, kinship, mixed model, cluster analysis

## Abstract

**Background:**

The inheritance of most human diseases and agriculturally important traits is controlled by many genes with small effects. Identifying these genes, while simultaneously controlling false positives, is challenging. Among available statistical methods, the mixed linear model (MLM) has been the most flexible and powerful for controlling population structure and individual unequal relatedness (kinship), the two common causes of spurious associations. The introduction of the compressed MLM (CMLM) method provided additional opportunities for optimization by adding two new model parameters: grouping algorithms and number of groups.

**Results:**

This study introduces another model parameter to develop an enriched CMLM (ECMLM). The parameter involves algorithms to define kinship between groups (that is, kinship algorithms). The ECMLM calculates kinship using several different algorithms and then chooses the best combination between kinship algorithms and grouping algorithms.

**Conclusion:**

Simulations show that the ECMLM increases statistical power. In some cases, the magnitude of power gained by using ECMLM instead of CMLM is larger than the improvement found by using CMLM instead of MLM.

**Electronic supplementary material:**

The online version of this article (doi:10.1186/s12915-014-0073-5) contains supplementary material, which is available to authorized users.

## Background

Genome-wide association studies (GWAS) are widely used in human genetics research to identify genes associated with complex diseases and in agricultural research to identify genes associated with quantitative traits such as yield and productivity [1,2]. The extremely dense genetic markers derived from new genotyping technology, such as genotyping by sequencing, have provided the potential for discovering genes underlying phenotypic diversity through GWAS [3-5]. Several new methods have been proposed for GWAS such as the multi-locus mixed-model approach [6] and the candidate gene approach [7]. However, automatically including cofactors, as proposed by these new methods, is challenging when the number of predictors is large compared to the number of observations. The model space is usually too large to explore exhaustively, and the maximum number of polymorphisms fitted at a time must be less than the number of individuals [6].

These issues are particularly problematic in recent GWAS because the number of polymorphisms can reach millions, but the number of phenotyped and genotyped individuals is rarely more than hundreds of thousands. For candidate gene studies, pre-requisite knowledge is necessary, for example, the location of the candidate genes. In this case, single-locus approaches are a necessary step before further analyses using the multi-locus or candidate gene approaches. Thus, the single-locus approach is still the mainstream method in GWAS.

However, advances in genotyping technology have allowed extremely dense genetic marker mapping and the associated computing time has become a major concern for genetic researches. Simultaneously, using large numbers of markers has also increased concerns about false positives [8-11] and the potential for misleading results in follow-up re-sequencing studies.

False positives are easy to control, but only at the expense of true positive discovery or statistical power. For example, a stringent association test threshold is an effective way to control the false positive rate, but the numbers of true positives are reduced at the same time. A desirable solution is to reduce false positives without compromising statistical power. This solution is critical because the inheritance of most human diseases and agriculturally important traits is controlled by many genes, which individually have small effects or rare alleles [12,13].

A number of statistical methods have been developed to eliminate spurious association between phenotypes and testing markers and to increase statistical power in GWAS. One of the causes of spurious association is population structure or stratification. In this case, the population can be partitioned into subpopulations. Then, association tests can be performed within the subpopulations or by using an estimate of population membership as a covariate in a linear or logistic model [14]. A similar method employs a principal components analysis of the genotype matrix. The first few principal components may reflect broad patterns of similarity across individuals [15-17].

Spurious association can also be caused by differences in relatedness between pairs of individuals. This effect can be reduced using a general linear model (GLM) to estimate the proportion of genes identical by descent between any pair of individuals and excluding closely related individuals [18,19]. Alternatively, population structure and unequal relatedness can be simultaneously accounted for in a mixed linear model (MLM). Subpopulation memberships (Q matrix) or principal components (PC) of the marker genotypes are treated as fixed effects and kinship is used to define the variance and covariance structure of random individual effects [20]. This MLM method outperforms other methods with respect to statistical power. An improved approach, called the compressed MLM (CMLM), has been proposed to cluster individuals into groups by using clustering algorithms such as the un-weighted pair group method with arithmetic mean (UPGMA). The kinship among groups is calculated simply as the average of kinship among individuals. By optimizing grouping (the number of groups and clustering method used for grouping), CMLM improves statistical power for GWAS [21].

This study introduces another parameter for model optimization: defining the relationship among groups in the CMLM. Statistical power is examined through simulations. The results showed that statistical power is further improved through this enriched compressed MLM (ECMLM) method.

## Results

### Model setup

With Henderson’s notation [22], a CMLM for GWAS can be written in as follows:

1 $$ \mathbf{y}=\mathbf{X}\boldsymbol{\upbeta } +\mathbf{Z}\mathbf{u}+\mathbf{e} $$

where **y** is a vector of a phenotype; **β** represents unknown fixed effects, including population structure and marker effects; **u** is a vector of size *s* (the number of groups) for unknown random polygenic effects following a distribution with mean of zero and covariance matrix of $\mathbf{G}=2\mathbf{K}{\sigma}_a^2$; and **K** is the group kinship matrix with element *k*_*ij*_(*i*, *j* = 1, 2,.... *s*) representing the relationship between group *i* and *j*, and ${\sigma}_a^2$ is an unknown genetic variance. **X** and **Z** are the incidence matrices for **β** and **u**, respectively, and **e** is a vector of random residual effects that are normally distributed with zero mean and covariance $\mathbf{R}=\mathbf{I}{\sigma}_e^2$, where **I** is the identity matrix and ${\sigma}_e^2$ is the unknown residual variance.

**Figure 1:**
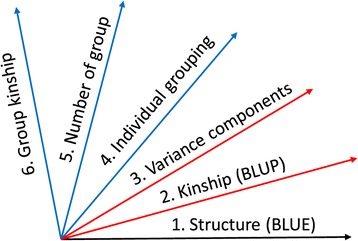
**Parameter space for association study.** The first dimension (in black) applies to both a general linear model and mixed linear model (MLM). The other dimensions apply to MLM only. The population structure (Structure) is fitted as a fixed effect with effect estimated as the best linear unbiased estimates (BLUE). The second dimension introduces individuals as random effects with variance defined by a kinship matrix. The best linear unbiased prediction (BLUP) for random effects can be solved directly with known variance components. The third dimension estimates unknown variance components using algorithms such as the residual maximum likelihood algorithm. The fourth dimension clusters individuals into groups (compression) by using cluster algorithms. The fifth dimension determines the best number of groups or average number of individuals per group (defined as compression level). The current study developed a sixth dimension that determines the best algorithm to define group kinship, for example, average, median, or maximum. The two dimensions in red belong to the standard MLM based on the individuals. The remaining dimensions (in blue) belong to the compressed MLM based on groups.

The likelihood of equation (1) can be expressed as:

2 $$ L\left(\mathbf{y}\left|\boldsymbol{\upbeta}, \mathbf{u},{\sigma}_a^2,{\sigma}_e^2,g,s,\phi \right.\right) $$

where **g** is a clustering algorithm; **s** is the number of groups; and *ϕ* is the operation used to calculate group kinship *k*_*ij*_ from individual kinship $\left({\tilde{k}}_{ht}\right)$. *ϕ* is the new parameter added in this study. The general formula for the derivation of pair-wise kinship coefficients is as follows:

3 $$ {k}_{ij}=\begin{array}{c}\hfill \phi \hfill \\ {}\hfill h\in i,t\in j\hfill \end{array}\left({\tilde{k}}_{ht}\right) $$

where the operation *ϕ* was defined as the average algorithm in the previous study [21]. Here we extended the operation to a series of algorithms, including average, median, and maximum. This extension created another dimension of parameter space in the MLM (Figure 1).We expected the extended parameter space would lead to a better model fit and result in higher statistical power for GWAS.

### Effect of group kinship algorithms in model fit

**Figure 2:**
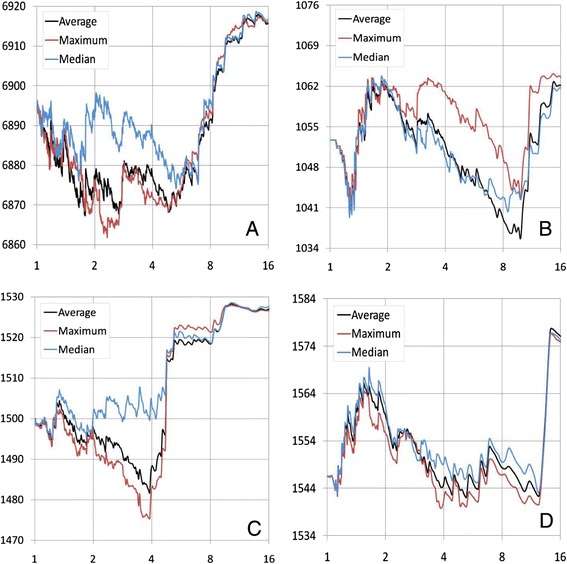
**Model fit of three different group kinship algorithms.** The model fit (vertical axis) is indicated by twice the negative likelihood (the smaller, the better). The grouping was performed with the un-weighted pair group method with arithmetic mean clustering algorithm at different compression levels (horizontal axis), defined as the average number of individuals per group. The phenotypes are **(A)** body mass index in human, **(B)** Orthopedic Foundation for Animals score in dog, and flowering time in both **(C)** maize and **(D)** *Arabidopsis*.

We examined model fit using three group kinship algorithms (average, median, and maximum) in four species (human, dog, maize, and *Arabidopsis*) where the UPGMA algorithm is used to cluster individuals into groups. The model fit was reflected by twice negative log likelihood (−2LL). Here, we define the compression level as the average individual number in each group. Different compression levels (up to 16 individuals per group on average) were applied. Variations of model fit due to each group kinship algorithm were observed for all species and at some compression levels between 1 and 16 (Figure 2). The average algorithm performed best only for the dog dataset. The maximum algorithm performed best for all other datasets. This finding suggested that optimization on group kinship algorithms is necessary for choosing the best algorithm for a specific dataset.

### Optimization on the extended parameter space

The joint use of group kinship and individual grouping enlarged the parameter space for the CMLM. We examined three group kinship algorithms (average, median, and maximum) and eight hierarchical clustering algorithms. The eight clustering algorithms were: UPGMA; un-weighted pair-group centroid (UPGMC); complete linkage (COM); Lance-Williams flexible-beta method (FLE); McQuitty’s similarity analysis, which is also called weighted pair-group method using arithmetic averages (WPGMA); weighted pair-group method using centroid (WPGMC); single linkage (SIN), which is also called nearest neighbor; and Ward’s method (WAR). Each combination was examined in the four species (human, dog, maize, and *Arabidopsis*).

**Figure 3:**
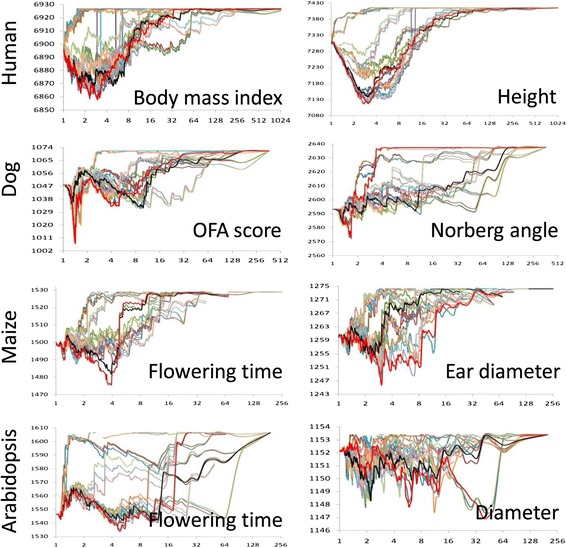
**Model fit of enriched compressed mixed linear model.** The model fit (vertical axis) is indicated by twice the negative log likelihood (−2LL). The model fit at different compression levels (horizontal axis) was examined for the 24 combinations (lines with different colors) between the three group kinship algorithms and the eight clustering algorithms. The combination in the standard compressed mixed linear model (average group kinship and un-weighted pair group method with arithmetic mean clustering algorithm) is labeled black. The rest of the combinations are labeled in color. The best combination (with the lowest -2LL) is labeled red. A better combination than the standard compressed mixed linear model was found in all the traits in the four species. OFA, Orthopedic Foundation for Animals.

Variation of model fit was observed at different compression levels (Figure 3). We found at least one combination with better model fit than the combination of UPGMA and the average group kinship algorithm used in the standard CMLM.

We previously examined 107 traits from *Arabidopsis* using the TASSEL software package [23]. We found three *Arabidopsis* traits for which the CMLM method failed to provide an advantage, based on model fit by using the average group kinship and the UPGMA clustering algorithm. The details are provided in Additional file 1. The three traits were aphid offspring number, visual chloros present at 16°C, and vegetative growth rate after vernalization. When the parameter space was expanded by the combinations of clustering methods and group kinship calculations, compression improved the fit for all three traits (Additional file 1: Figure S1). Therefore, extension of the parameter space improved the performance of CMLM.

### Computing time

ECMLM effectively increases the potential to balance statistical power and computing speed. When the goal was to have statistical power equivalent to standard MLM, enriched compression resulted in much higher compression levels. Because computing time is a cubic function of the compression level, enriched compression greatly reduced computing time (Additional file 1: Table S1). For the human dataset, the number of groups was reduced from 166 to 33 (a five-fold reduction). The observed computing time was reduced from 8.89 seconds to 0.73 seconds (a 12-fold decrease).

When conducting GWAS, we first optimized the model without markers using population parameter previously determined (P3D) to find the best compression level, kinship, and cluster algorithms. This process took 80 minutes (InterCore2 Duo CPU E7500, 2.93GHz, Memory 1.99G) to perform ECMLM using *Arabidopsis* data containing 199 lines and 5000 SNPs. The CMLM took 3.5 minutes to finish this step, but used only one combination between kinship algorithm and clustering algorithm. Compared to the CMLM, the ECMLM method requires additional time to optimize population parameters, depending on the number of algorithm combinations tested. However, ECMLM finds the optimal combination of compression level, kinship, and cluster algorithms, resulting in higher statistical power and a better model fit. The optimized parameter values can then be used for SNP association testing, which is the most time-consuming step in GWAS.

### Statistical power and false positive control of association study

The statistical power of a method corresponds to model fit. We compared the statistical power of the ECMLM method with three other methods: GLM, MLM, and CMLM. The ECMLM was performed using the best of 24 combinations between the three group kinship algorithms and the eight clustering algorithms that cluster individuals into groups across all compression levels. The CMLM reported previously used the average group kinship and the UPGMA clustering algorithm to cluster individuals into groups across all compression levels. MLM and GLM used the minimum and the maximum compression levels, respectively. Each individual was treated as a single group in the MLM. All individuals were clustered as one group (merged into the overall mean) in the GLM. In these two cases, both the clustering algorithm and the group kinship algorithm would have no effect.

**Figure 4:**
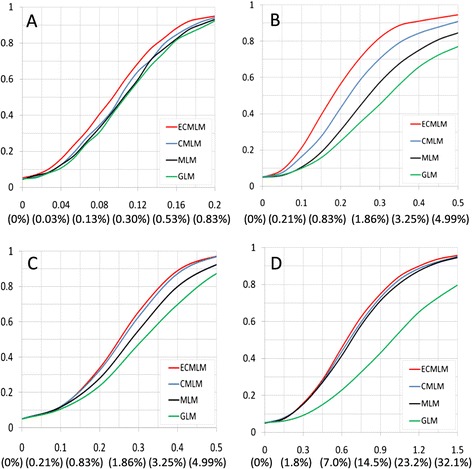
**Statistical power of four statistical methods.** The four methods are general linear model (GLM), mixed linear model (MLM), compressed MLM (CMLM), and enriched compressed MLM (ECMLM). The CMLM was performed with the un-weighted pair group method with arithmetic mean clustering algorithm and the average algorithm for calculating group kinship. The ECMLM was performed by the best combination of three group kinship algorithms and eight clustering algorithms. The statistical power was evaluated on a simulated phenotype with the quantitative trait nucleotide (QTN) effect added to observed phenotypes. The size of the QTN effect is expressed in the unit of phenotypic standard deviation. The corresponding proportions of total phenotypic variance explained are displayed in the parentheses. The observed phenotypes are **(A)** body mass index in human, **(B)** Orthopedic Foundation for Animals score in dog, and flowering time in both **(C)** maize and **(D)** *Arabidopsis*.

The statistical power was estimated empirically by adding quantitative trait nucleotides (QTNs) to an observed phenotype, then calculating the proportion of detected QTNs. The threshold was determined from the distribution of *P*-values on the observed phenotype before the QTN effect was added. The observed phenotypes are body mass index in human, Orthopedic Foundation for Animals (OFA) score in dog, and flowering time in both maize and *Arabidopsis*. Statistical power improvements with the ECMLM method were observed compared to other methods (Figure 4). Improvements of up to 6.4%, 13.3%, 2.9%, and 2.6% were observed when the ECMLM was compared to the CMLM in human, dog, maize, and *Arabidopsis*, respectively (Additional file 1: Table S2). Based on the human dataset, the improvement in statistical power from CMLM to ECMLM was larger than the improvements from GLM to MLM or from MLM to CMLM.

**Figure 5:**
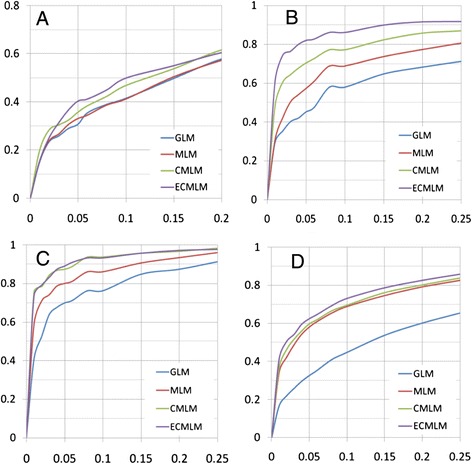
**Statistical power under different false discovery rates for four statistical methods.** The four methods are general linear model (GLM), mixed linear model (MLM), compressed MLM (CMLM), and enriched compressed MLM (ECMLM). The CMLM was performed with the un-weighted pair group method with arithmetic mean clustering algorithm and the average algorithm for calculating group kinship. The ECMLM was performed by the best combination of three group kinship algorithms and eight clustering algorithms. The statistical power was evaluated on a simulated phenotype with the quantitative trait nucleotide (QTN) effect added to observed phenotypes. The size of the QTN effect is expressed in the unit of phenotypic standard deviation (SD). The x-axis indicates the false discovery rate; the y-axis shows statistical power. The observed phenotypes are **(A)** body mass index in human with SD = 0.08, **(B)** Orthopedic Foundation for Animals score in dog with SD = 0.3, and flowering time in both **(C)** maize with SD = 0.4 and **(D)** *Arabidopsis* with SD = 0.75.

Statistical power under the same false discovery rate (FDR) was also examined for these four methods. The size of the QTN effect is expressed in the unit of phenotypic standard deviation (SD). The observed phenotypes are body mass index in human with SD = 0.08, OFA score in dog with SD = 0.3, and flowering time in both maize with SD = 0.4 and *Arabidopsis* with SD = 0.75 (Figure 5). We examined the power under different FDR levels. At the same FDR levels, the ECMLM method performed better than the other three methods in all datasets, especially in the dog data. So, the ECMLM can control the FDR while improving statistical power.

Comparison of statistical power for the four models (GLM, MLM, CMLM, and ECMLM) using different numbers of PCs was also investigated (Additional file 1: Figure S2). The comparison used from one to five PCs to control the population structure. The ECMLM was performed using the best combination of the three group kinship algorithms and eight clustering algorithms. The statistical power was evaluated on a simulated phenotype with the QTN effect added to observed phenotypes. The size of the QTN effect is expressed in the unit of phenotypic standard deviation. The observed phenotype is the flowering time of *Arabidopsis* at 10°C. We found that different PCs have little effect on statistical power in MLM, CMLM, and ECMLM.

## Discussion

The ECMLM method adds a new parameter to the existing CMLM GWAS method by examining alternatives to calculating kinship between groups as the average of pair-wise individual kinships. This new parameter brings the total number of parameter types in the model to six. The first parameter type is the population structure fitted as a variable number of fixed effects. The second (random genetic effect) and the third (variances or ratio) parameters relate to the standard MLM. The last three parameters relate to CMLM. Two of them - clustering methods and number of groups - were investigated in a previous study. The current study focused on a sixth parameter: ways of defining group kinship (Figure 1). Similar to each of the first five dimensions, the sixth dimension also improves statistical power.

The essential element in cluster analysis is to define the similarity between groups. Many clustering algorithms are named based on the property of similarity. Consequently, group kinship algorithms share the footprints from the individual clustering algorithms. For example, with the maximum algorithm, the kinship between two groups is defined as the maximum kinship between an individual in one group and an individual in another group. Therefore, the maximum algorithm is named the single linkage in cluster analysis, also called nearest neighbor. Single linkage possesses the best theoretical properties [24].

The opposite of single linkage is complete linkage clustering (furthest neighbor method), which sets the similarity between two groups equal to the smallest similarity between an individual of one cluster and an individual of another cluster. This method tends to produce very tight clusters of similar cases and corresponds to using the minimum algorithm. The minimum algorithm gave no advantage over others on the data examined and, therefore, was excluded from this study.

In the average method, the kinship between two groups is the average of the all the individual pair-wise kinships between the groups. The average method used in the CMLM corresponds to the average linkage in cluster analysis [25]. Average linkage is also known as the UPGMA.

The median method in the CMLM does not correspond to any typical clustering method. Instead of using the median, the centroid is commonly used for cluster analysis. Among the three group kinship algorithms we investigated, the median algorithm never performed the best for any trait from the four species. The other two algorithms (average and maximum) switched back and forth, competing for the best in conjunction with clustering algorithms to group individuals.

Future studies that test other clustering algorithms are needed. We only examined eight (UPGMA, UPGMC, COM, FLE, WPGMA, WPGMC, SIN, and WAR) of many algorithms that cluster individuals into groups. For example, we did not test any non-hierarchical clustering algorithms (for example, K-means); all clustering algorithms used in this study are hierarchical clustering algorithms.

We found a huge variation in model fit among the various combinations of the three group kinship algorithms and the eight clustering algorithms. In general, the optimum combinations were trait- and species-specific (Figure 3). Optimization is necessary for each specific dataset.

The improvement in model fit by introducing a sixth parameter is accompanied by improvement in statistical power for GWAS. Some improvements are large. The improvement in statistical power achieved by using ECMLM on the human dataset instead of CMLM is greater than the power gained by using MLM instead of GLM or by using CMLM instead of MLM (Figure 4 and Additional file 1: Table S2).

The computing time for optimization on the extended parameter space increased linearly with the number of algorithms in the new dimension. This increase becomes irrelevant in GWAS with many markers by using P3D. The optimization only needs to be performed once for all the parameters in the model except SNP. Those parameter values can then be used for tests on SNP associations.

For the compression level corresponding to the best model fit, some of the extended parameter space have a higher compression level for some traits and lower for others. We found no indication whether the extension would increase or decrease compression level for best model fit. However, if the objective is to reach the same model fit as the standard MLM, the extended parameter space could dramatically increase compression level. Because the computing time is cubic to the compression level, a huge improvement in computing time could be realized using ECMLM under such an objective (Figure 3). If the objective is to achieve the same compression level or computing speed as MLM, higher statistical power could be realized by using the ECMLM method.

The combined usage of group kinship algorithms and clustering algorithms for grouping individuals created an extended parameter space for GWAS. The extension of parameter space made more traits suitable for the application of compression to improve statistical power in GWAS (Additional file 1: Figure S1).

## Conclusion

The enrichment of the compressed mixed linear model by optimizing group kinship improves statistical power for genome wide association studies. The enriched compressed mixed linear model is applicable on more wide range of complex traits.

## Methods

### Observed data

Four datasets from human, dog, maize, and *Arabidopsis* were examined in this study. Each dataset contained phenotype data and a set of genetic markers. All the datasets have been described in previous studies [20,21,26-28], including the distribution of kinship elements derived from the genetic markers. The human dataset was collected from 1,315 adult individuals (European Americans over 17 years old) who participated in the Genetics of Lipid Lowering Drugs and Diet Network (GOLDN) study [29]. The dataset included 647 genetic markers (388 microsatellite, or simple sequence repeat (SSR), and 259 SNP markers) scored on the individuals. All multi-allelic SSR markers were converted into bi-allelic markers by collapsing alleles into two states: major alleles and all other alleles. Measured phenotypes included height and body mass index.

The dog dataset was sampled from a dataset used for mapping quantitative trait loci underlying canine hip dysplasia [27,28] and a dataset used to estimate heritability of canine hip dysplasia [30]. The dataset contained 292 dogs from two breeds (Labrador Retriever and Greyhound) and their crosses (F_1_, F_2_, and two backcrosses). Hip dysplasia was measured as the Norberg angle (a measure of hip congruency) and the OFA hip score. All dogs were genotyped with 23,500 SNPs at genome-wide coverage and 1,000 SNPs were randomly sampled for this study.

The maize dataset was composed of phenotypes, genotypes (553 SNPs), and a population structure (Q matrix) calculated for 277 inbred lines [20]. The phenotypes included flowering time scored as days to pollination and ear diameter.

The *Arabidopsis* dataset included 199 landraces genotyped by 216,130 SNPs [26]. We randomly sampled 5,000 SNPs for this study. Among the 107 available traits, two traits (flowering time at 10°C and plant diameter at flowering) with the fewest missing observations were chosen to study model fit and statistical power.

### Statistical power estimation

We added a QTN effect to the observed phenotype. We assigned the QTN to each marker, one at a time. The resulting simulated phenotypes retained the original genetic architecture, such as population structure relatedness [20]. The proportion of detected QTNs was used as the empirical estimate of statistical power. An SNP was considered a detected QTN if the association test statistics passed a threshold. The genotypic effect of each marker was fit as a fixed effect. The association tests on the markers’ genotypes were performed by F tests. The threshold was determined by the empirical distribution of the F statistics on the original observed phenotype before the artificial QTN effect was added. The *P*-value at the bottom 5% quantile was used as the empirical threshold [20].

The QTN effect was represented in the unit of phenotypic standard deviation. The percentage of the total variation explained by the QTN (π) is a function of allele substitution difference (d) and sample frequency (*p*) of the polymorphism at the QTN: π = 1/(1 + 1/p(1-p)d^2^) [31]. Different effects were added for human (a maximum of d = 0.2), dog (a maximum of d = 0.5), maize (a maximum of d = 0.5), and *Arabidopsis* (a maximum of d = 1.5) according to sample sizes. To facilitate comparison between datasets, we listed π at the allele frequency of *p* = 0.3. The genetic effect was assigned to all SNPs, one at a time, to produce replicates across all SNPs.

### Statistical analysis

Observed and simulated phenotypes were analyzed using Proc Mixed in SAS [32]. Variance components were estimated with the restricted maximum likelihood algorithm. For human, the fixed effects were sex, age, and the quadratic term of age. Similarly, breed (or fraction of Labrador Retriever for the crosses with Greyhound) was the fixed effect for dog, and population structure was the fixed effect for maize and *Arabidopsis*. Population structure was represented by the fractions of subpopulation in maize using Structure software. The population structure of *Arabidopsis* was represented by the first two PCs derived from the SNPs. Previous study indicates that models incorporating both structure and kinship perform better than when including them separately [20].

Individuals or their corresponding groups were fit as a random effect. The kinship among individuals was estimated from the genetic markers by the approach of Loiselle *et al*. [33]. The individuals in each dataset were grouped based on their kinship using Proc Cluster in SAS [32]. Eight hierarchical clustering algorithms [34] were examined: UPGMA, UPMGC, COM, FLE, McQuitty’s similarity analysis (WPGMA), WPGMC, SIN, and WAR. At different compression levels, −2LL was used to compare model fit.

### Data availability

ECMLM has been implemented in GAPIT (R package). Source code and support documents (user manual, demo data, demo script, and demo results) are available at GAPIT [35].

### Ethics statement

All the datasets analyzed here were from previously published datasets. This study did not involve taking actual samples from humans or animals.

## Additional file

Additional file 1:
**Additional Figure S1-S2 and Table S1-S2.**

## Abbreviations

-2LL: Twice negative log likelihood
CMLM: compressed mixed linear model
COM: complete linkage
ECMLM: enriched compressed mixed linear model
FDR: false discovery rate
FLE: Lance-Williams flexible-beta method
GLM: general linear model
GOLDN: Genetics of Lipid Lowering Drugs and Diet Network
GWAS: genome-wide association study
MLM: mixed linear models
OFA: Orthopedic Foundation for Animals
P3D: population parameter previously determined
PC: principal component
QTN: quantitative trait nucleotides
SIN: single linkage
SNP: single nucleotide polymorphism
SSR: simple sequence repeat
UPGMA: un-weighted pair-group centroid
UPGMC: un-weighted pair-group centroid
WAR: Ward’s method
WPGMA: weighted pair-group method using arithmetic averages
WPGMC: weighted pair-group method using centroid
