# Supplementary material for: Enrichment of statistical power for genome-wide association studies
Source: BMC Biol. 2014 Oct 17;12:73. doi: 10.1186/s12915-014-0073-5 (PMC4210555; doi:10.1186/s12915-014-0073-5)
Supplement: Additional file 1: — Additional Figure S1-S2 and Table S1-S2. [file 12915_2014_73_MOESM1_ESM.docx]

Support document

1.
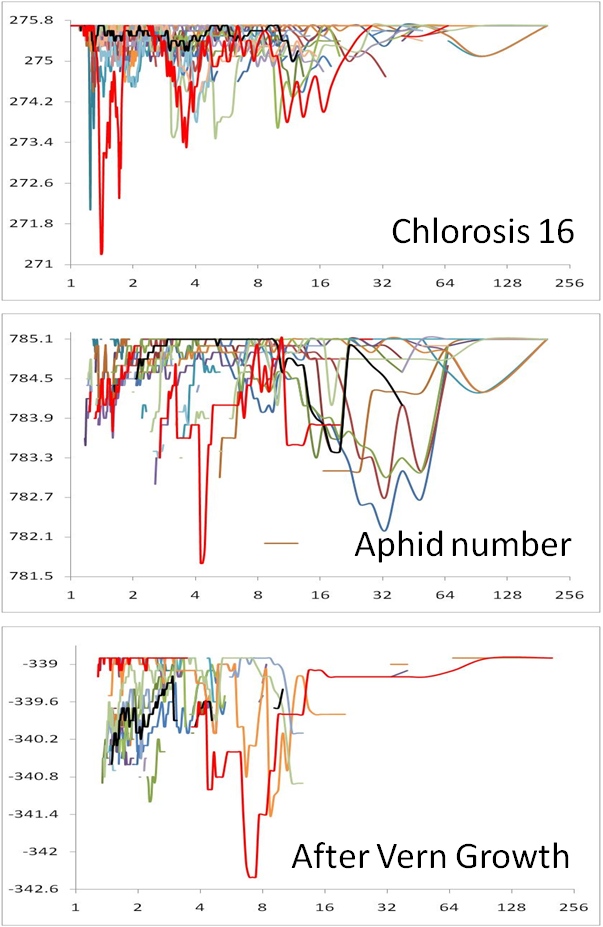

2. **Figure S1.**Model fit for the 3special traits(out of 107)in *Arabidopsis*. These three traits were the only ones that did not show improvement on model fit through compression performed by the TASSEL software package. The three traits were Chlorosis16 (chlorosis presence at 16℃),, Aphid (offspring) number, and After Vern Growth (vegetative growth rate after vernalization).The compression with TASSEL was performed with the average group kinship algorithm and UPGMA clustering algorithm on a subset of compression levels defined as average number of individuals per group. The screen on the full set of compression levels with the Enriched compression clearly showed the improvement of model fit for these three traits. The model fit (vertical axis) is indicated by twice the negative log likelihood (-2LL). The model fit at different compression levels (horizontal axis) was examined for the 24 combinations (lines with different colors) between the 8group kinship algorithms and the 8clustering algorithms. The combination in the standard compressed MLM (average group kinship and UPGMA clustering algorithm) is labeled as black. The rest are in colors. The best combination (with the lowest -2LL) is labeled as red. A better combination than the standard compressed MLM was found for all the three traits.


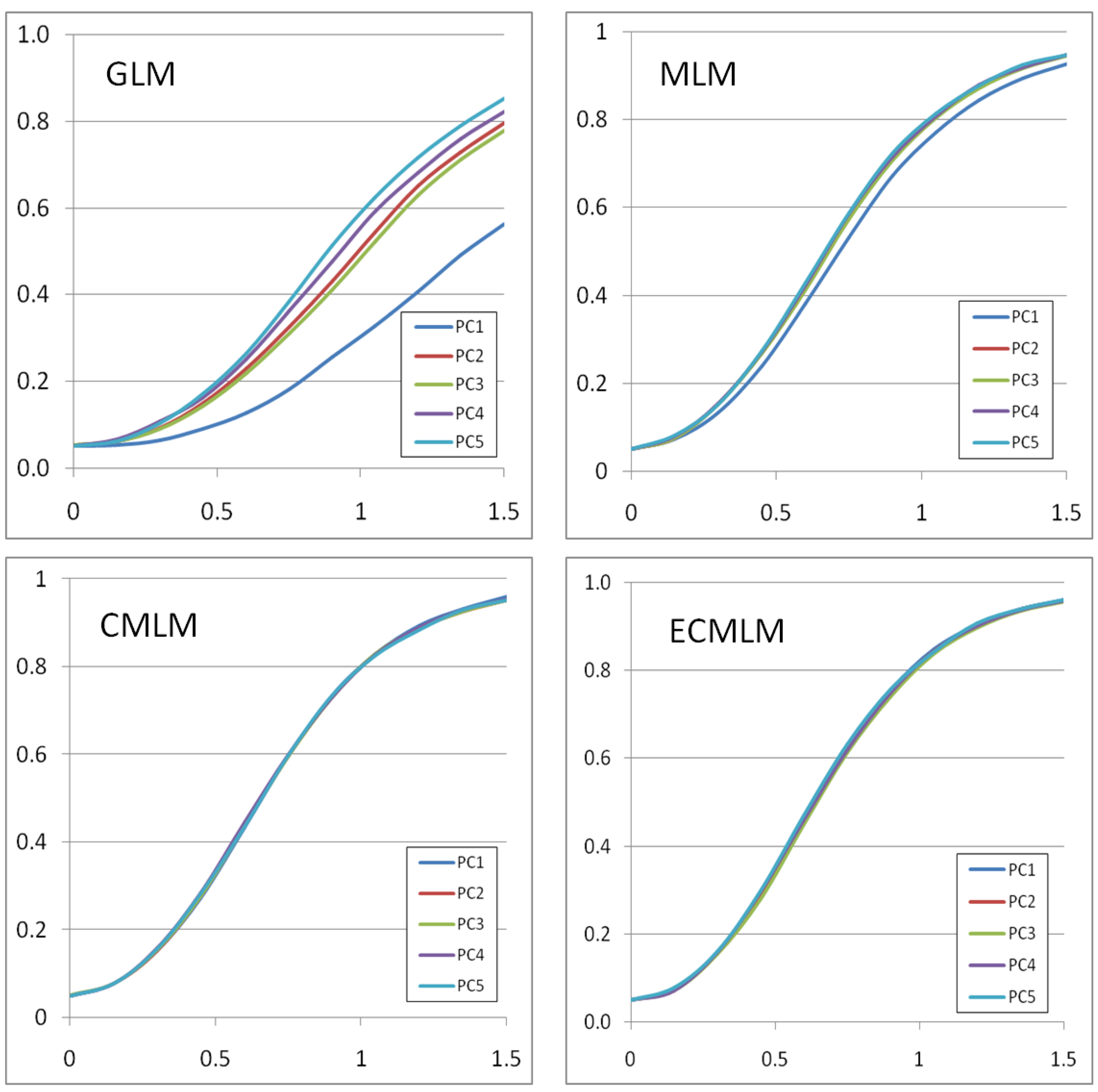


**Figure S2.** Comparison of power for 4 model including different number of PCs. The model used different PC number from one to five to control the population structure. Four methods are employed to perform the comparison, generalized linear model (GLM), mixed linear model (MLM), compressed mixed linear model(CMLM) and enriched compression mixed liner model (ECMLM). The ECMLM was performed by the best combination of three group kinship algorithms and eight clustering algorithms. The statistical power was evaluated on a simulated phenotype with the QTN effect added to observed phenotypes. The size of the QTN effect is expressed in the unit of phenotypic standard deviation. X axis is the added deviation and Y axis shows the power. The observed phenotype is the flowering time at 10℃ of *Arabidopsis*.

**Table S1.** Computing time to perform a single association analysis.

| **Priority** | **Method** | **Human** | **Dog** | **Maize** | **Arabidopsis** |
| --- | --- | --- | --- | --- | --- |
| Not available | MLM | 866.05 (1315) | 12.08(366) | 2.27(277) | 1.75(199) |
| Model fit | CMLM | 168.32(736) | 0.38(37) | 0.38(71) | 0.16(41) |
|  | ECMLM | 57.19 (447) | 8.40(259) | 0.38(71) | 0.14(41) |
| Speed | CMLM | 8.89(160) | 0.30(34) | 0.28(59) | 0.06(16) |
|  | ECMLM | 0.73(33) | 0.13(9) | 0.17(31) | 0.04(3) |

1. The unit of computing time is second. The association analysis was performed by using mixed linear model (MLM) with optimization of variance components. The compressed MLM used the average group kinship and chose the optimum algorithm from the eight cluster algorithms to group individuals. The enriched compression used the optimum combination from the 24 combinations of the 3group kinship and 8clustering algorithms. The priority of the optimization was set as: 1) model fit which selected the combination and the compression level corresponding to the best model fit; 2) speed which selected the maximum compression level with model fit that was equivalent to, or above, the one from the standard MLM. The numbers in parentheses are the number of groups clustered. Each individual was treated as a group in the standard MLM.

**Table S2**. Increases of statistical power in three advances of statistical methods.

| **Method advance** | **Human** | **Dog** | **Maize** | **Arabidopsis** |
| --- | --- | --- | --- | --- |
| GLM to MLM | 3.6% | 13.8% | 10.1% | 29.6% |
| MLM to compression | 4.0% | 14.2% | 7.6% | 2.5% |
| Compression to Enriched compression | 6.4% | 13.3% | 2.9% | 2.6% |

The increase was calculated as the maximum difference between two methods across different magnitude of QTN effect in each species. For example, for a QTN (quantitative trait nucleotide) contributing 0.3% of total phenotypic variation, the statistical power was increased from 67.8% by using general linear model (GLM) to 71.4% by using mixed linear model (MLM) with a increase of 71.4% -67.8%= 3.6%.
